# Hepatitis C Virus p7 is Critical for Capsid Assembly and Envelopment

**DOI:** 10.1371/journal.ppat.1003355

**Published:** 2013-05-02

**Authors:** Juliane Gentzsch, Christiane Brohm, Eike Steinmann, Martina Friesland, Nicolas Menzel, Gabrielle Vieyres, Paula Monteiro Perin, Anne Frentzen, Lars Kaderali, Thomas Pietschmann

**Affiliations:** 1 Institute of Experimental Virology, TWINCORE, Centre for Experimental and Clinical Infection Research; a joint venture between the Medical School Hannover (MHH) and the Helmholtz Centre for Infection Research (HZI), Hannover, Germany; 2 Institute for Medical Informatics and Biometry, Medical Faculty, University of Technology Dresden, Dresden, Germany; University of California, San Diego, United States of America

## Abstract

Hepatitis C virus (HCV) p7 is a membrane-associated ion channel protein crucial for virus production. To analyze how p7 contributes to this process, we dissected HCV morphogenesis into sub-steps including recruitment of HCV core to lipid droplets (LD), virus capsid assembly, unloading of core protein from LDs and subsequent membrane envelopment of capsids. Interestingly, we observed accumulation of slowly sedimenting capsid-like structures lacking the viral envelope in cells transfected with HCV p7 mutant genomes which possess a defect in virion production. Concomitantly, core protein was enriched at the surface of LDs. This indicates a defect in core/capsid unloading from LDs and subsequent membrane envelopment rather than defective trafficking of core to this cellular organelle. Protease and ribonuclease digestion protection assays, rate zonal centrifugation and native, two dimensional gel electrophoresis revealed increased amounts of high-order, non-enveloped core protein complexes unable to protect viral RNA in cells transfected with p7 mutant genomes. These results suggest accumulation of capsid assembly intermediates that had not yet completely incorporated viral RNA in the absence of functional p7. Thus, functional p7 is necessary for the final steps of capsid assembly as well as for capsid envelopment. These results support a model where capsid assembly is linked with membrane envelopment of nascent RNA-containing core protein multimers, a process coordinated by p7. In summary, we provide novel insights into the sequence of HCV assembly events and essential functions of p7.

## Author Summary

Viroporins are small hydrophobic viral membrane proteins which oligomerize and modulate membrane properties to facilitate virus propagation. Within their membrane environment these proteins can form membrane pores or channels which change the permeability of membranes for ions. These properties are known to contribute to release of infectious enveloped virus particles from infected cells and/or to facilitate viral cell entry by catalyzing virus uncoating. In case of HCV, p7 function is essential for production of infectious progeny and its ion channel activity is well documented *in vitro* and in cell-based systems. Recent evidence indicated that p7 channel activity dissipates the low pH of the cellular secretory compartment thus protecting the viral glycoproteins from low pH induced misfolding and inactivation. In this investigation we highlight the involvement of the p7 ion channel in the assembly and envelopment of viral RNA-containing capsids. Our results indicate that p7, likely in concert with the viral envelope proteins, comprises a membrane-bound recipient complex that provides a scaffold to initiate unloading of core protein from lipid droplets for capsid assembly and membrane envelopment. Collectively, these findings highlight novel facets of p7 function in the course of HCV morphogenesis.

## Introduction

Approximately 160 million people are chronically infected with HCV [1] and viral persistence is associated with severe liver diseases such as steatosis, cirrhosis and hepatocellular carcinoma [2]. HCV is an enveloped virus with a positive-strand RNA genome that encodes a polyprotein of about 3,000 amino acids. The polyprotein is cleaved co- and post-translationally by cellular and viral proteases into 10 mature proteins. Briefly, the structural proteins are resident in the N-terminal portion of the polyprotein and include the core protein and the envelope glycoproteins E1 and E2.The nonstructural proteins NS3, 4A, 4B, 5A and 5B are encoded on the C-terminal part of the polyprotein, and are components of the viral replicase complex [3]. Finally, the p7 and NS2 proteins reside in between structural proteins and NS3-5B. Both are dispensable for replication but necessary for the production of progeny viruses [4], [5], [6].

The pathways of HCV morphogenesis including capsid assembly, particle envelopment and virus egress are incompletely understood. Moreover, there is limited information about the structure and composition of HCV particles as well as the precise association of the viral structural proteins and accessory factors during virus production. However, recent work mostly based on the JFH1-based HCV infection model [7], [8], [9] has highlighted some unique features of the late steps of the HCV life cycle. Firstly, lipid droplets, cellular lipid storage organelles, have been recognized to be essential for production of infectious HCV progeny [10]. Core protein resides on the surface of these lipid storage organelles [11], [12] and during virus assembly recruits viral proteins and RNA, which is an essential prerequisite for virus production [10]. Core protein itself is loaded onto these lipid bodies through an interaction with diacylglycerol acyltransferase-1 (DGAT-1) [13], a host enzyme which catalyzes the final step in the biosynthesis of triglycerides that is essential for lipid droplet biogenesis [14]. Moreover, host factors crucial for the biosynthesis and secretion of human lipoproteins have emerged as cofactors for HCV morphogenesis. These include apolipoprotein B (ApoB), apolipoprotein E (ApoE) and microsomal triglyceride transfer protein (MTTP) [15], [16], [17]. Presumably as a consequence of the tight coupling of HCV assembly with lipoprotein biogenesis, infectious HCV is thought to circulate as so called “lipo-viro particle”, rich in cholesteryl esters and comprising viral proteins, ApoB and ApoE [18], [19], [20]. Finally, it is recognized that besides the viral structural proteins (core, E1, E2) and p7, essentially all NS proteins contribute to production of infectious viral progeny [4], [5], [21], [22], [23], [24], [25], [26]. Yet, how each of these proteins contributes to infectious HCV particle assembly and release is currently poorly defined.

It is thought that HCV core proteins form the viral capsid which encases the viral RNA. Monomeric, mature core protein has a molecular weight of 21 kDa and exerts dual affinity for RNA and for lipids. Based on a hydropathicity profile, the N-terminal domain 1 (D1) is hydrophobic and extends to residue 117 [27]. It mediates core protein homo-oligomerization [28], [29], [30], [31] as well as its interaction with the viral RNA [32], [33], [34]. The latter seems to be poorly sequence-specific and to depend primarily on the presence of structured RNA [29], [30], [35]. Domain 2 (D2, ca. residues 118–174 [27]) is on the contrary hydrophobic and composed of two amphipathic α-helices connected by a hydrophobic loop [36]. This domain is responsible for the localization and anchorage of the mature core protein at the surface of lipid droplets [37]. Finally, domain 3 (D3) is composed of the C-terminal portion of core and acts as a signal sequence for insertion of E1 into the ER lumen. Notably, an interaction between core and the E1 envelope protein has been proposed [38], [39]. The envelope shell harbors the E1 and E2 glycoproteins, whose heterodimers appear to be crosslinked by disulfide bridges [40]. The NS5A protein, more specifically the C-terminal domain III, is also essential for production of infectious HCV [25], [41], [42]. Interestingly, phosphorylation of specific serine residues within this domain of the protein has been implicated in virus production, suggesting a model where NS5A phosphorylation governs replication and assembly [42]. Moreover, accumulating evidence from several independent studies supports a key role of NS2 in coordinating assembly by interacting with the glycoprotein complex, but also with p7, NS3 and NS5A thus bringing together the different viral components for envelopment [43], [44], [45], [46]. While NS2 function has been studied extensively the precise role of p7 in this process remains unclear.

P7 is a small membrane protein of 63 aa with both termini oriented towards the ER lumen. It contains an N-terminal α-helix followed by two transmembrane (TM) domains connected by a cytosolic loop (aa 33–39) [47]. Importantly, p7 monomers can assemble into hexamers or heptamers [48], [49], [50] and thereby form cation-selective ion channels in planar lipid bilayers, thus classifying p7 as a viroporin [48], [51], [52], [53]. A fully conserved di-basic motif within the cytoplasmic loop (residues K33 and R35 for genotype 2a J6) is important for the channel function in artificial membranes as well as for virus assembly and release in cell culture [4], [48], [54]. Finally, the essential role of p7 was also confirmed in vivo [55]. Notably, recent evidence suggested that p7 acts as a proton channel in the secretory compartment of the cells and dissipates the low pH in these compartments to protect low pH-sensitive nascent particles and envelope proteins [54]. However, it was unclear if this was the sole function of p7 during HCV assembly. Therefore, in this study we developed a set of complementary biochemical and cell biological methods to dissect the individual steps involved in HCV morphogenesis in order to shed further light on the specific function(s) of p7 in the course of infectious virion morphogenesis.

## Results

### Characterization of HCV constructs with mutations in structural proteins or p7

**Figure 1:**
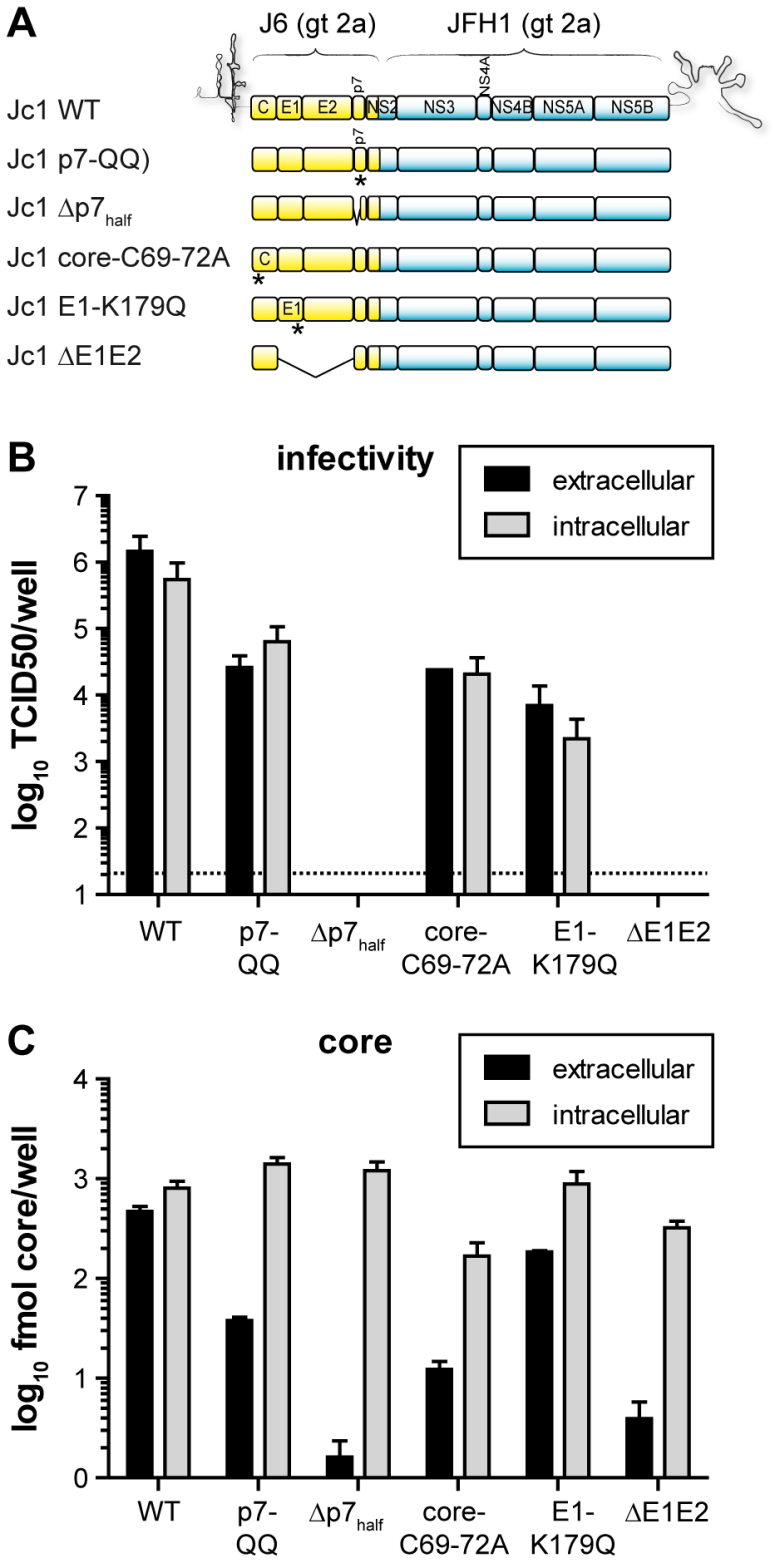
Specific mutations in HCV structural proteins and p7 impair virus assembly. (A) Schematic representation of the HCV constructs used in the study. All constructs were derived from the genotype 2a chimera Jc1. Point or deletion mutants in HCV structural proteins served as references throughout the study for the p7-QQ and Δp7_half_ mutants. (B,C) Huh7-Lunet-hCD81-Gluc cells were transfected with WT-Jc1 or mutant RNA. After 48 h, cell supernatants were harvested and intracellular lysates were prepared by repetitive cycles of freeze and thaw. Intra- and extracellular infectivity was determined by TCID_50_ titration on Huh-7.5 cells (B) and core levels were quantified by ELISA (C). In panel B, the dotted line indicates the detection threshold of the assay. Mean values and standard deviations of three independent experiments are shown.

To investigate the precise function of p7 during HCV morphogenesis, several p7 mutants with published defects in virus production were utilized, among them a point mutant of the dibasic motif in the loop region (p7-KR33/35QQ, or p7-QQ [4]) abrogating ion channel activity, and a more severely impaired deletion mutant lacking the predicted p7 N-teminal transmembrane helix (p7 residues 1 to 32) (Δp7_half_, [4]). For comparison, various genomes with mutations of one or more structural proteins were characterized in parallel. These included a mutant carrying four consecutive alanine residues in place of amino acids 69 to 72 of J6CF-core (core-C69-72A [56]), and a construct with a mutation in the transmembrane region of E1, known to disturb E1–E2 heterodimerization and virus production (E1-K179Q [4], [57]). Finally, we also created a mutant which lacks the entire coding region of E1 and E2 (ΔE1E2) (Figure 1A). Note that all mutants were generated in the context of the highly infectious Jc1 chimeric HCV genome [58]. To verify and compare the influence of these mutations on HCV assembly and release of infectious virions, extra- and intracellular virus titers in HCV-transfected cells were determined with a limiting dilution assay (Figure 1B). While the point mutations similarly reduced secreted virus titers ca. 100-fold, virus production was completely abrogated for the two deletion constructs. Furthermore, the parallel decrease in intracellular infectivity strongly argues for a specific impairment of HCV assembly rather than release. Only for the p7-QQ mutant, extracellular infectivity was slightly more reduced compared to intracellular infectivity, suggesting the possibility of an additional block in virus secretion. To rule out that the changes observed were indirectly caused by an effect of the mutations on HCV RNA replication, translation or specific infectivity of virus particles, extra- and intracellular levels of core protein were determined by ELISA (Figure 1C). As expected, we observed comparable intracellular core amounts for all virus variants indicating that the assembly defects were not due to disturbed RNA replication or translation (Figure 1C). For most mutants the decrease in extracellular infectivity correlated with a reduction in extracellular core levels. Therefore, the decreased extracellular infectivity is attributable to reduced assembly of infectious virions rather than secretion of poorly infectious, aberrant particles. Notably, this was not the case for the E1-K179Q mutant virus, for which core release was comparable to WT Jc1 despite a ∼100-fold reduction in extracellular infectivity titers (Figure 1B and C). This data suggests the secretion of non- or poorly infectious particles in case of this mutant. In conclusion, with the exception of E1-K179Q, all mutants clearly showed defects in HCV assembly with downstream effects on the number of particles released and the total intracellular and secreted infectivity. Moreover, the residual titers were comparable among point and deletion constructs, facilitating the comparison between these groups of mutants.

### Virus production of p7 mutants is blocked before core protein envelopment

**Figure 2:**
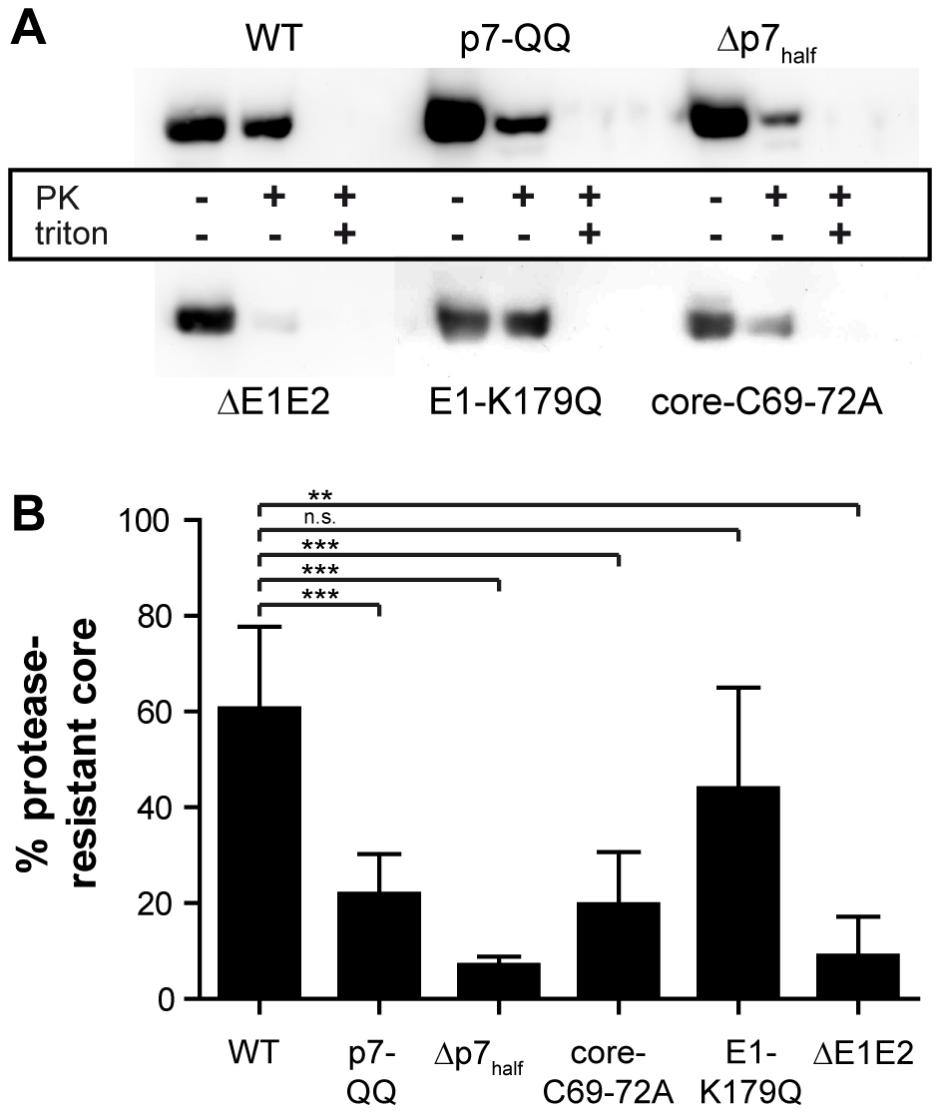
Assembly of the p7 mutants is impaired prior to capsid envelopment. WT-Jc1- or mutant-transfected detergent-free cell lysates were subjected to a proteolytic digestion protection assay as follows. Lysates were separated into three aliquots which received different treatments: (i) left untreated, (ii) treated with 50 µg/ml proteinase K for 1 h on ice, or (iii) lysed in 5% Triton X-100 prior to proteinase K treatment (condition used for background correction). The amount of protease-resistant core was determined by Western Blot and ELISA. (A) Representative Western Blot stained for HCV core. (B) Western Blot signal intensities were quantified with LabImage 1D and values obtained for the proteinase K-treated sample were background-corrected and normalized to untreated control. Mean values and standard deviations of 3–6 independent experiments are shown.

To pinpoint the specific defect(s) in HCV morphogenesis caused by these mutations, we dissected the assembly process into distinct steps such as core trafficking to the lipid droplets (LDs), core oligomerization, capsid formation, and, finally, envelopment. First of all, core protein resistance to proteinase K digestion was assessed as a measure of the degree of envelopment of the protein into membranes, which is expected to protect the protein from proteolytic digestion. Briefly, cells transfected with mutant or wild-type (WT) Jc1 were lysed mechanically and treated with proteinase K. Residual core protein was quantified by immunoblotting or ELISA and normalized to the total amount of core protein in the untreated sample. As a control, cell lysates were pretreated with Triton X-100 to solubilize all membranes before proteinase K digestion. Under these latter circumstances, all core proteins are expected to be sensitive to proteolytic digestion, ensuring that the proteinase K concentration was not limiting in the assay. The core amount detected under these conditions was used for background subtraction. A representative immunoblot stained for HCV core is shown in Figure 2A. In the context of WT-Jc1, quantification of signal intensities revealed that about 60% of intracellular core protein was resistant to protease digestion and thus, had already acquired a membrane envelope and proceeded to a post-budding step (Figure 2B). In contrast, all mutants with the exception of E1-K179Q, which was not significant (p = 0.084, Welch's t-test), showed a highly significant (p<0.01, Welch's t-test) reduction in amount of protected core, indicating that most core species present were digested. Together, these data suggest that the mutations/deletions under investigation confer a defect in infectious particle production occurring at, or prior to, core envelopment.

### P7 mutants accumulate non-enveloped capsid-like structures

**Figure 3:**
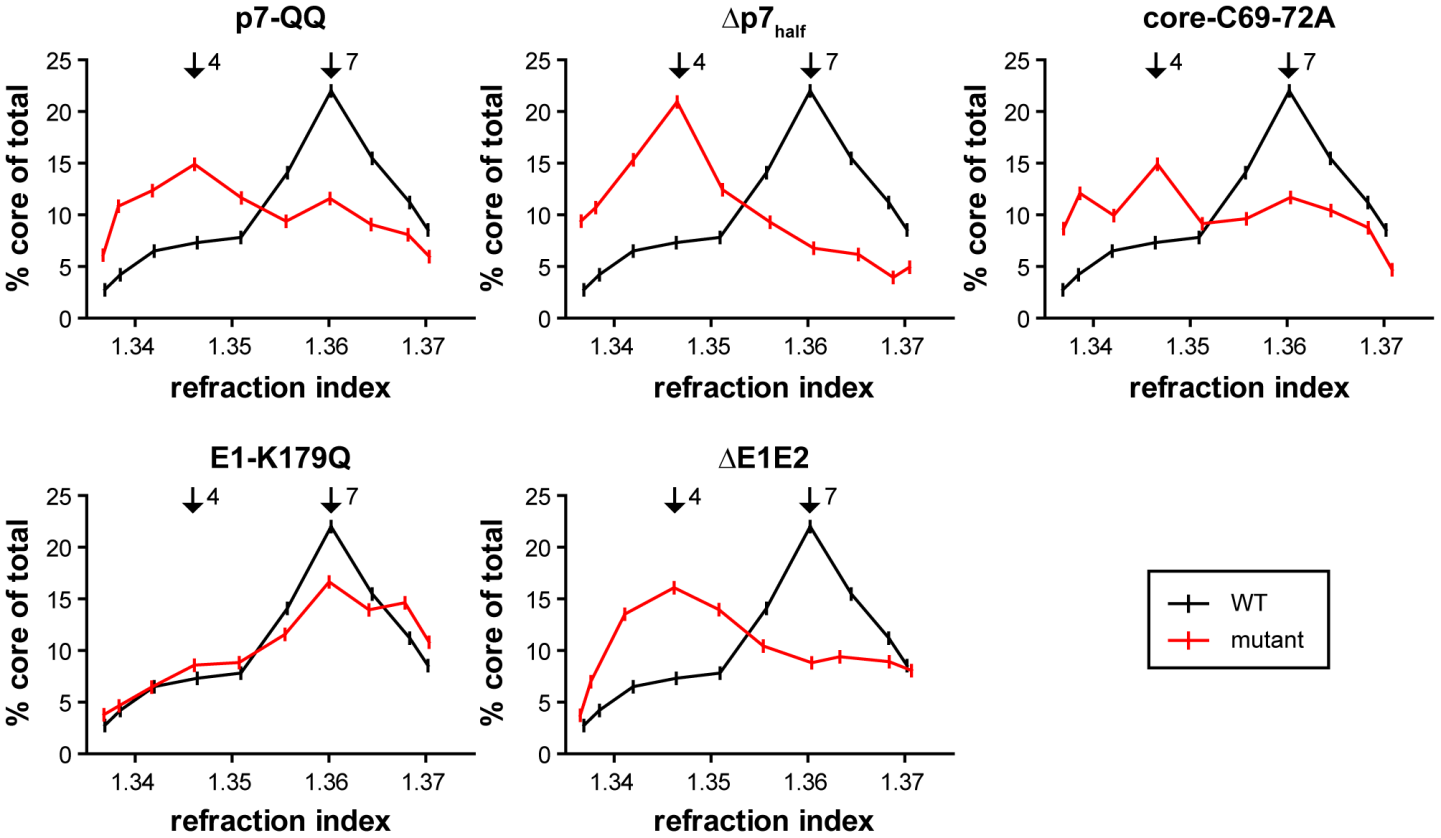
Rate zonal separation of core protein complexes in absence of detergent. Postnuclear supernatants of cell lysates obtained by repetitive cycles of freeze and thaw 48 h post-transfection were layered on top of a preformed continuous 0–30% sucrose density gradient and subjected to rate zonal centrifugation for 1 h at 270,000× g. Core content was measured along the gradient by ELISA and normalized to the total core amount in the lysate. Mean values of 3–5 independent experiments are plotted. Fractions 4 and 7 are highlighted with black arrows.

**Figure 4:**
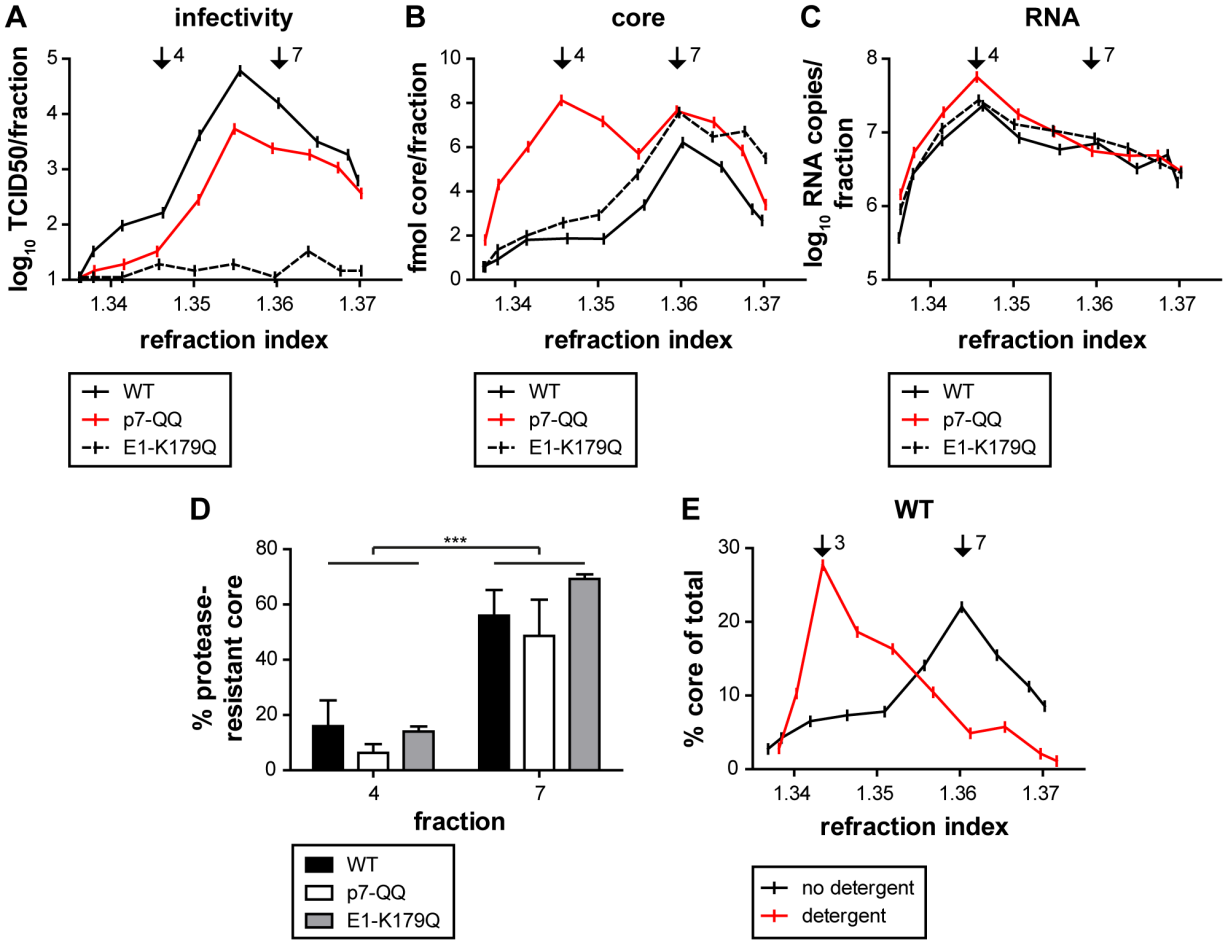
Characterization of differentially-sedimenting core complexes. Lysates of cells transfected with WT or mutant Jc1 were separated by rate zonal density gradient centrifugation. Each fraction was analyzed for infectivity by TCID_50_ (A), core protein content by ELISA (B), and RNA copy number by qRT-PCR (C). Fractions 4 and 7 are highlighted with black arrows. (D) Fractions 4 and 7 were subjected to a proteolytic digestion protection assay and the amount of protease-resistant core protein was quantified by ELISA. The values obtained for the proteinase K-treated samples were background-subtracted and normalized to the untreated controls. Mean values and standard deviations of 2 independent experiments are shown. (E) Lysates of WT Jc1-transfected cells were separated by rate zonal density gradient centrifugation in the absence or presence of 1% DDM and core distribution along the gradient was determined by ELISA.

With the reduced amounts of membrane-protected core observed, we next analyzed if this was due to a defect in capsid envelopment itself or due to blockade of events prior to this. To address this question, HCV-transfected-cells were lysed by multiple cycles of freeze and thaw. Subsequently, post-nuclear lysates were separated by rate zonal centrifugation in the absence of detergent. The core protein content of each fraction was determined by ELISA and normalized to the total core amount in the lysate. While there was a single prominent peak of rapidly sedimenting core in fraction 7 with a refraction index of 1.360 for WT-Jc1 and the E1-K179Q mutant (Figure 3), the other mutants clearly showed a second peak, sedimenting more slowly at fraction 4 with a refraction index of around 1.345. In fact, in the case of the deletion constructs and p7-QQ, core protein species with reduced mobility were the predominant species. Notably, expressing core protein from a replicon RNA lacking E1, E2, p7 and NS2 caused a similar accumulation of core protein in fraction 4 (Supplementary Figure S1). In order to further characterize different core protein species, including those forms with slower-sedimentation, HCV infectivity and RNA content were determined in each gradient fraction. Interestingly, the core peak in fraction 7 which is predominant in the case of WT-Jc1 was highly infectious (Figure 4A). In contrast, fraction 4, which displays the accumulation of core protein that is characteristic for the p7 mutants and the deletion constructs (Figure 3 and Figure 4B) contained relatively little infectivity: 100-fold reduction compared to fraction 7. (Figure 4A). Since fraction 4 of the WT-Jc1 preparation contained only 3-fold less core protein than fraction 7 (Figure 4B) and as most of the viral RNA sediments in fraction 4 (Figure 4C), it is unlikely that the dramatic reduction of infectivity is attributable to lack of viral RNA or core protein. Of note, the sedimentation of viral RNA in the gradient was independent of core protein expression, since distribution of viral RNA after transfection of a Jc1 mutant lacking most of the core coding region (Jc1- Δcore) was comparable to Jc1-WT transfected cells (Supplementary Figure S2). Given these findings we suspected that core protein species sedimenting in fraction 4 may represent incomplete, non-enveloped, particles. In contrast, fraction 7 carries a substantial amount of infectious particles. Notably, the fraction with highest infectivity in the sedimentation was fraction 6. This suggests that either fraction 7 besides infectious virions carries an excess of non-infectious viral RNA and core compared to fraction 6 or that some HCV particles may “mature” causing differential sedimentation (fraction 6) and higher infectiousness. To address the observed differences between fractions 4 and 7, we subjected these fractions to proteolytic digestion by protease K treatment. Interestingly, only approximately 10–20% of core protein was protected from proteolysis in fraction 4 whereas about 50–60% of core protein was protease-resistant in fraction 7, giving a highly significant difference in the two fractions (p = 3.909e-5 over all mutants, Figure 4D). Notably, the degree of core protein protection specific to fraction 4 and 7 was similar between WT-Jc1, the E1 and the p7 mutant, indicating that all three genomes yield comparable core protein structures – albeit with different efficiency. Moreover, specifically the E1 mutant displayed very low infectivity throughout all fractions (Figure 4A), which may be due to a defect of particles carrying this mutation in cell entry. Altogether, these results indicate that fully-enveloped infectious intracellular HCV particles preferentially sedimented in fraction 7 and that the p7 mutant is heavily impaired in producing core protein species with these sedimentation properties. Strikingly, supplementation of the rate zonal gradient with detergent to strip core protein from lipid envelope led to a shift in core sedimentation of WT-Jc1 from fraction 7 to fractions 3 in the low density range of the gradient (Figure 4E). These results indicate that non-enveloped capsids and/or oligomeric core protein liberated after detergent treatment preferentially sediment in fractions 3of the detergent-carrying gradient. Therefore, mutations/deletions in p7 can increase accumulation of non-enveloped capsids and/or oligomeric core proteins which sediment in fraction 4 of our rate zonal gradient (in the absence of detergent). It is possible that these structures may be similar to true capsids released from intracellular WT-Jc1 particles which sediment in fraction 3 of the detergent gradient.

### Production of enveloped intracellular core structures is impaired and delayed for HCV with defective p7

**Figure 5:**
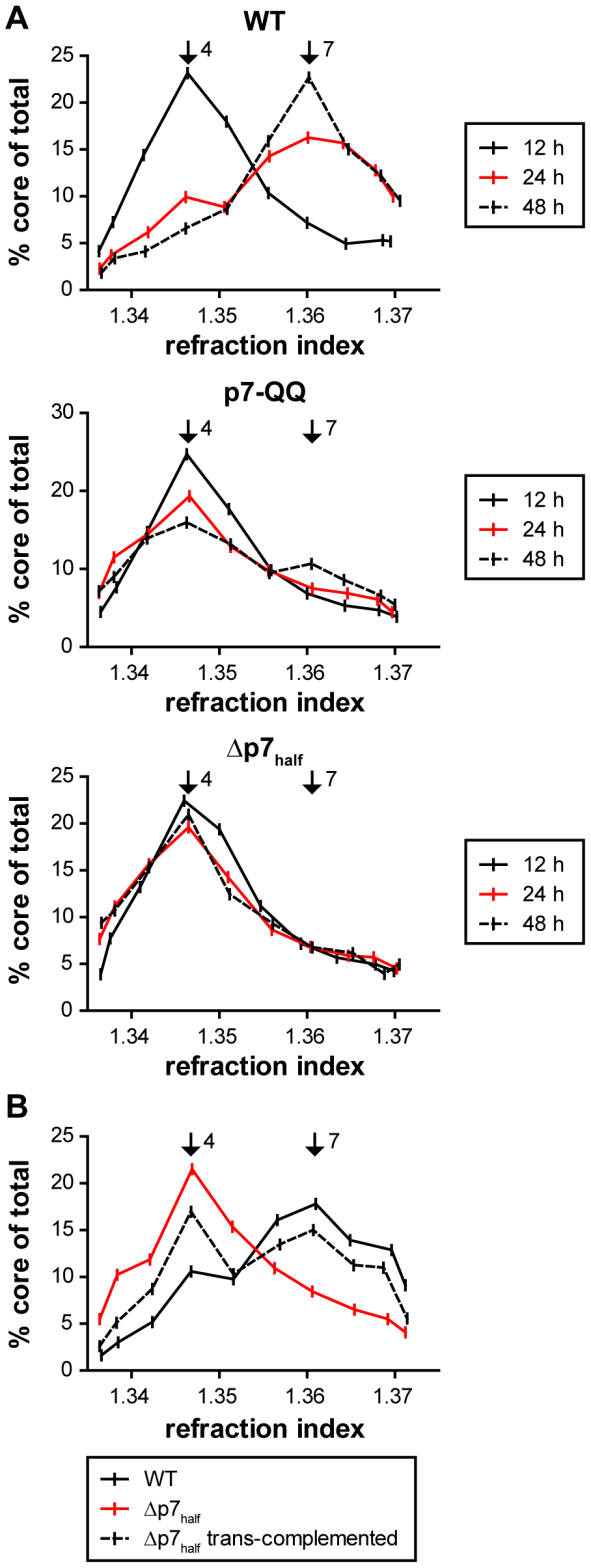
Kinetics of capsid envelopment and rescue of p7 defects by trans-complementation. (A) Cell lysates were harvested at the indicated time points post-transfection and subjected to rate zonal density gradient centrifugation. Core distribution along the gradient was determined by ELISA. (B) Jc1 Δp7_half_ RNA was transfected into either naïve Huh-7.5 cells or packaging cells stably expressing the core to NS2 region. As a comparison, WT-Jc1 RNA was transfected into naïve Huh-7.5 cells. Lysates were harvested 24 h post-transfection, subjected to rate zonal density gradient centrifugation and the core distribution in the gradients was analyzed by ELISA. Fractions 4 and 7 are indicated with black arrows.

To further investigate the natural course of capsid envelopment, HCV transfected cells were collected at 12, 24 and 48 h after transfection and subjected to rate zonal centrifugation as described above (Figure 5A). Interestingly, at an early time point (12 h post-transfection), the sedimentation profile of core protein was identical for WT-Jc1 and the p7 mutants, with a peak at fraction 4, similar to the one observed 48 h post-transfection for the p7 mutants (Figure 3 and Figure 5A). While this peak remained unchanged over time for the Δp7_half_ mutant, a fraction of core protein displayed increased mobility as early as 24 h post-transfection and sedimented in fraction 7 in the case of WT-Jc1. Finally, at 48 h almost all core protein in the WT-Jc1 transfected cells sedimented in fraction 7. An intermediate phenotype was observed for the p7-QQ mutant, which exhibited a delayed and incomplete shift in core sedimentation profile. This change in mass and/or size of core complexes over time, evidenced here by different rate zonal centrifugation profiles, likely reflected capsid envelopment, a process apparently impaired or abrogated respectively for the p7-QQ and Δp7_half_ mutants. Therefore, early during assembly, capsid envelopment and possibly budding represent a rate-limiting step that involves p7. We next investigated whether this p7 function could be complemented in *trans* and thus, transfected Jc1 Δp7_half_ RNA into a packaging cell line expressing the core to NS2 region [59]. Strikingly, transfection of the p7 mutant into the packaging cell partially restored infectivity (data not shown). Concomitantly, the core sedimentation profile was partially shifted back to enveloped core species with rapid sedimentation (Figure 5B) indicating that the defect in membrane envelopment could be at least partially rescued in *trans*.

### Defective p7 causes accumulation of core protein at the surface of lipid droplets

**Figure 6:**
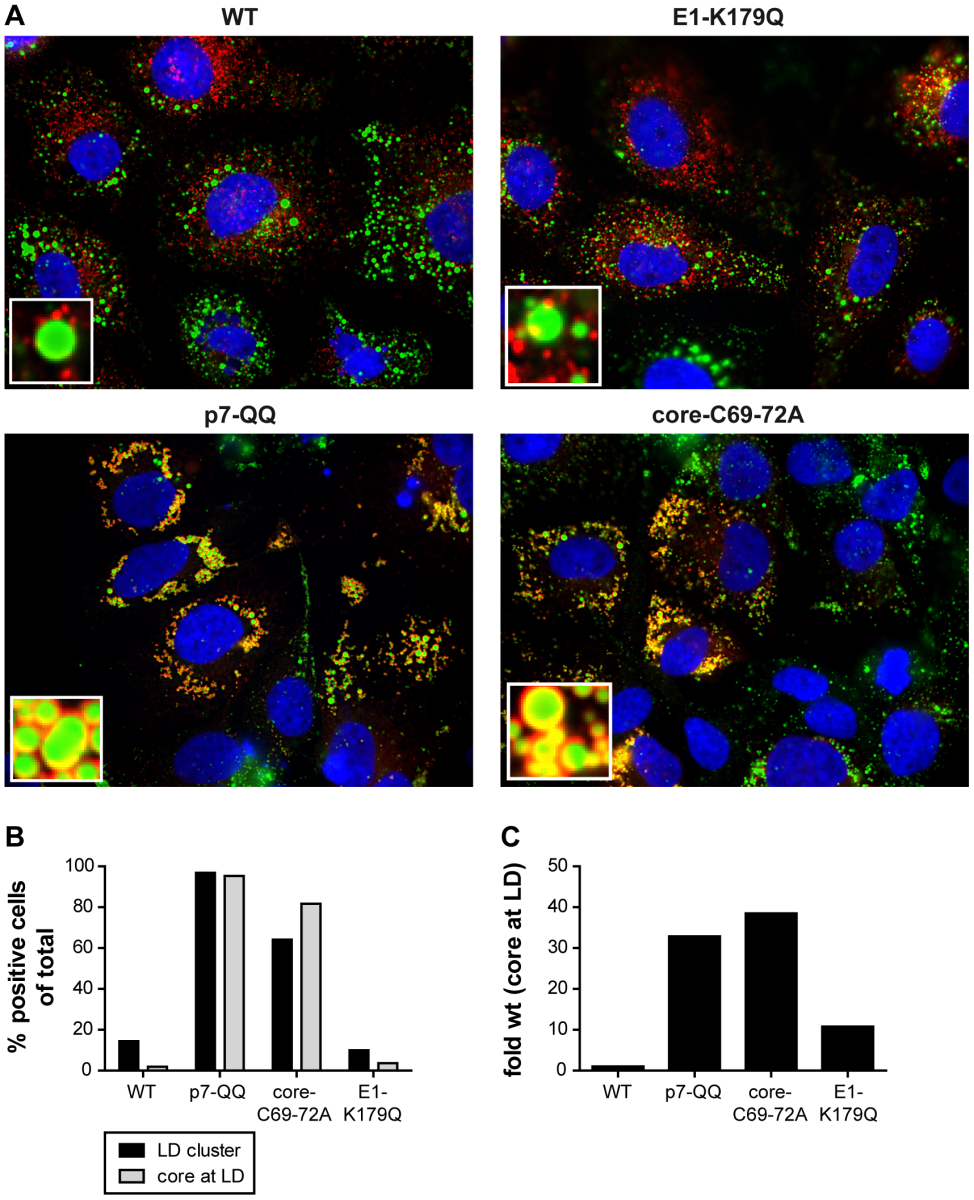
Retention of core protein at the surface of lipid droplets. (A, B) WT-Jc1 or mutant-transfected cells were fixed after 48 h and subjected to immunofluorescence staining of core (C7-50 antibody, red) and LD (BODIPY, green). Nuclei were counterstained with DAPI (blue). Representative pictures are shown in A. Small panels show magnifications of the LD area. (B) At least 100 transfected cells were counted for each construct and assessed for LD clustering and core accumulation at LDs. (C) Lipid droplet isolation. HCV-transfected cells were lysed by Dounce homogenization in a hypotonic medium. Lipid droplets were separated by flotation through a discontinuous density gradient. Core protein amounts were measured by ELISA, normalized to the total core content in the lysate and the fold difference in comparison to WT-Jc1 was calculated.

All assembly mutants tested, with the exception of the E1-K179Q mutant, showed an abnormal accumulation of non-enveloped capsids or core oligomers (Figure 3). To further explore the molecular mechanisms underlying this defect, we next compared the subcellular localization of core protein between Jc1-WT, E1-K179Q, p7-QQ and the core-C69-72A mutants by immunofluorescence staining using core-specific antibodies and co-staining of lipid droplets by the lipid dye BODIPY (Figure 6A). As published previously in the context of WT-Jc1, core protein demonstrated a reticular staining pattern with relatively little LD association [60], [61]. Moreover, LDs were well dispersed throughout the cell. The same phenotype was observed for E1-K179Q (Figure 6A). In contrast, accumulated core protein was located at the surface of clustered lipid droplets in the case of p7-QQ and core-C69-72A mutants (Figure 6A). To quantify these differential phenotypes, we analyzed between 100 and 200 cells for each mutant and assigned HCV expressing cells into two categories characterized by elevated LD clustering (i.e. aggregation of at least 10 LDs in close proximity to each other) or increased LD association of core protein (circular core staining around the surface of LDs; Figure 6B). In the case of WT-Jc1 and the E1-K179Q mutant, only about 10% of cells showed LD clustering and even less cells displayed core staining around the entire surface of LDs. Strikingly, the frequency of both phenotypes was strongly increased for the p7-QQ and the core-C69-72A mutants with more than 90% of p7-QQ-transfected cells being positive for both (Figure 6B). To further confirm these observations, lipid droplets were isolated from cell lysates by flotation through a discontinuous density gradient. Enrichment of ADRP (a LD marker) and exclusion of calreticulin (a typical ER-resident protein) from the LD fraction confirmed the efficiency of the cell fractionation (data not shown). The amount of core protein associated with the LD fraction was determined by ELISA and normalized to the total core content, with the value obtained for WT-Jc1 set as 1 (Figure 6C). An over 30-fold increase in the amount of LD-associated core could be observed for the p7 and the core mutants whereas for E1-K179Q only ca. 10-fold more core protein was detected at LDs. Therefore, the enrichment of non-enveloped capsids for the p7 mutants (and the core-C69-72A mutant) correlates with an accumulation of core at the surface of lipid droplets. These findings strongly argue that these mutations cause the retention of core protein and possibly core oligomers and higher order capsid-like structures at those organelles.

### Defective p7 increases proportion of incompletely assembled capsids

**Figure 7:**
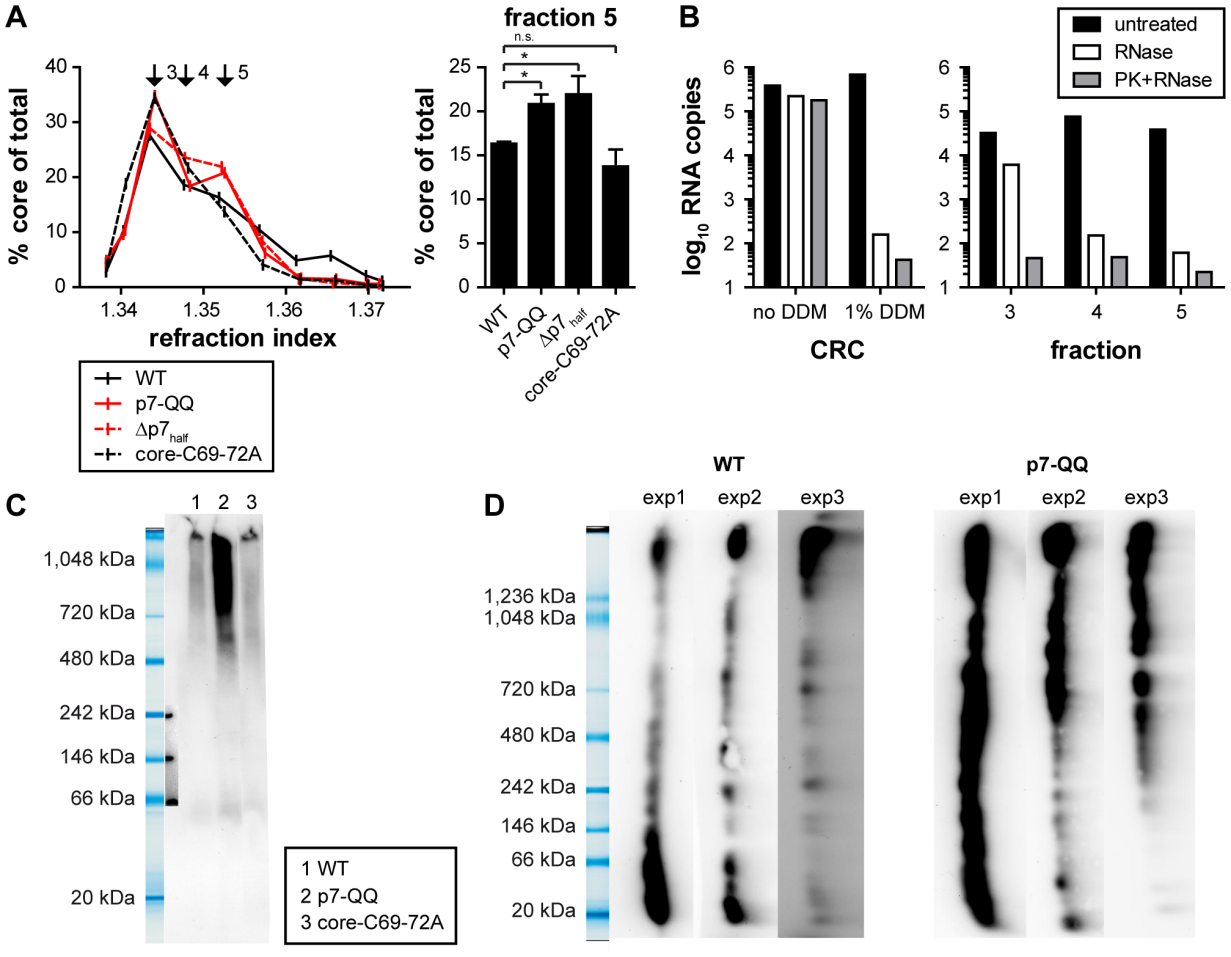
Mutations in p7 affect capsid assembly. (A) WT-Jc1 or mutant-transfected cells were harvested 48 h post-transfection. Cell lysates were subjected to rate zonal density gradient centrifugation in the presence of non-ionic detergent (1% DDM) and core protein amounts along the gradient were measured by ELISA. Fractions 3 to 5 are highlighted with black arrows. The right panel shows the core amount relative to total core expression for fraction 5. (B) RNase digestion protection assay (see Materials and Methods). Fractions 3 to 5 of the WT-Jc1 gradient were subjected to RNase digestion or left untreated. As a background control, samples were treated with proteinase K to remove protecting capsids, prior to RNase treatment. The amount of residual HCV RNA was quantified by qRT-PCR. In parallel crude HCV replication complexes (CRCs) were prepared by the method described by Quinkert et al. [62]. CRCs were then subjected to RNase treatment as described above. To mimic conditions of the sedimentation 1% DDM was added. (C) Separation of core complexes by blue-native-PAGE (4–16% gradient gel). (D) Two-dimensional gel electrophoresis of core complexes. Cell lysates were separated by blue native-PAGE (3–12% gradient gel) in a first electrophoresis and by SDS-PAGE in the second dimension. The results of three independent experiments are shown side by side. Gel pictures are shown vertically for easier comparison with the blue-native-PAGE results. (C, D) Core protein was detected by Western Blotting and subsequent immunodetection with the core-specific monoclonal antibody C7-50.

To further investigate if the observed defects in capsid envelopment were caused by prior alterations in capsid assembly, core complexes from HCV-transfected cell lysates were separated by rate zonal centrifugation in the presence of detergent. As observed before, the dominant core species from WT-Jc1 transfected, detergent treated cells sedimented in fraction 3 (Figure 7A). These core proteins likely represent mostly assembled capsids since the highly infectious enveloped core species from fraction 7 were converted to this sedimentation behavior by detergent treatment (Figure 4E). An indistinguishable profile was observed for the core-C69-72A mutant indicating that its inefficient envelopment was not caused by a defect in capsid assembly, as far as it can be resolved with this method (Welch's t-test against WT, Fraction 5: p = 0.3046, no significant difference). In contrast, a moderate, but significant (see right panel of Figure 7A) enrichment of slightly faster sedimenting core complexes was detected for both p7 mutants (fraction 5: p7QQ: p = 0.01556, Δp7half: p = 0.0417).

To further characterize these putative capsid assembly intermediates (or aberrant capsid species), we tested their ability to protect the viral RNA from RNase digestion (Figure 7B). To this end, gradient fractions 3 (corresponding to the core peak obtained with the WT-Jc1 construct), 4 and 5 (specifically enriched in the p7 mutants) were subjected to RNase digestion and residual viral RNA was quantified by qRT-PCR. To control the efficiency of the RNase digestion, RNA-protecting capsids or core oligomers (or other proteins) were removed by proteinase K pretreatment, before RNase addition. Strikingly, the viral RNA in fraction 3 was mostly protected from RNase digestion, unless the samples were treated with proteinase K beforehand. In contrast, RNase digestion strongly reduced HCV RNA levels in fraction 5, and pretreatment with proteinase K only marginally increased RNA degradation. Comparable results were also obtained for p7-QQ, Δp7_half_ and core-C69-72A mutants (Supplementary Figure S3). Notably, HCV RNA contained in crude replication complexes prepared from Jc1-WT transfected cells according to the protocol by Quinkert et al. [62] was almost completely protected from RNase digestion even when protease K was added (Figure 7B). However, when 1% DDM was added to these CRCs to mimic conditions of our rate zonal gradient, addition of RNase A alone was sufficient to degrade more than 99.9% of viral RNA. Collectively, these results argued that a large proportion of RNA complexes sedimenting in fraction 3 in the presence of 1% DDM are protected from ribonuclease digestion unless protease K was added. Since HCV RNA in CRCs does not share this property and is readily degraded even in the absence of protease, we believe it is unlikely that the RNA structures in fraction 3 predominantly represent CRCs. In contrast, due to the high abundance of core protein in fraction 3 (Figure 7A) and the observation that enveloped capsids are shifted to this fraction 3 (Figure 4E) we believe that the viral RNA in fraction 3 may be encased in a capsid-like structure thus protecting it RNase digestion. On the contrary, core complexes sedimenting in fraction 5 and enriched in mutant-transfected cells, might be loosely associated with the viral RNA and form incomplete, loose or unstable capsid structures that do not fully protect the RNA. Notably, we cannot dismiss the alternative interpretation that other proteins differentially sedimenting in fractions 3, 4 and 5 may cause a differential protection of the viral RNA to ribonuclease digestion.

Finally, in order to gain further insights about the size and oligomerization status of the various core complexes observed, lysates of HCV-transfected cells normalized for equal levels of HCV core were separated by native PAGE using 4-16% gradient gels with a resolution from 15 to 1,000 kDa. Strikingly, a large amount of core protein was detected as part of high-order complexes between 480 and 1,048 kDa for the p7-QQ mutant (Figure 7C) whereas for Jc1-WT and the core-C69-72A mutant, little core protein signal was visible. Possibly, most of the core protein could not enter the gel for WT-Jc1 and the core-C69-72A mutant, indicating that it belonged to large structures exceeding 1,000 kDa, presumably the fully-assembled capsids. Alternatively, the antibody used for detection (C7-50), may not recognize the predominant core species for these constructs. To circumvent this problem, two-dimensional gel electrophoresis was performed with native polyacrylamide gel electrophoresis (PAGE) in the first dimension and denaturing, reducing SDS-PAGE in the second. With this setup, all core complexes are dissociated into monomers during the second dimension of the electrophoresis thus avoiding possible problems caused by masking of the antibody epitope in native core protein complexes. Notably, the mobility of core in the gel reflects oligomerization status since the first dimension of the gel is resolved under native conditions. A 3–12% gradient gel was used in the first dimension to further broaden the resolution of the assay. Subsequent immunoblotting of core essentially confirmed the data obtained from the native PAGE (Figure 7D). Due to the inherent variability of the assay, three independent experiments are shown side by side for comparison. A variety of core complexes, ranging from monomeric to high-order structures, could be detected for WT-Jc1, however, the most prominent species exceeded the resolution of the gel and, thus, probably corresponded to fully-assembled capsids. In contrast, an enrichment of diverse intermediate high-order core complexes could be seen for the p7-QQ mutant.

In summary, the p7 mutants showed an accumulation of putative capsid assembly intermediates which may not have fully encased the viral RNA, while contrastingly the core C69-72A mutation interfered specifically with a downstream envelopment and/or budding event. Taken together, our results suggest that the presence of functional p7 is required for a late step of capsid assembly as well as capsid envelopment suggesting a tight linkage between these two processes.

## Discussion

Accumulating evidence supports the crucial role of non-structural proteins in HCV assembly and release [63] and in particular the essential part played by the p7 viroporin [4], [5]. Here, we unraveled the precise role of p7 in production of HCV progeny virions by characterizing the defective viral particle production of two p7 mutants, a double mutant impaired in its ion channel activity (p7-KR33,35QQ) [4], [48], and a more extreme deletion mutant lacking the first transmembrane domain (Δp7_half_) [4]. These mutants were studied in the context of the genotype 2a chimera Jc1, chosen for its high assembly efficacy [58], [60], allowing us to biochemically dissect the sequence of events involved in this process and to better resolve potential points of interference.

In a Jc1 background, we observed a ca. 60-fold reduction, or a complete block in infectious particle production, respectively for the p7-QQ and Δp7_half_ mutants (Figure 1B). Intracellular core levels of these mutants were unchanged (Figure 1C), ruling out gross RNA replication defects for both mutants. In case of p7-QQ the specific infectivity of released particles was comparable to WT-Jc1 thus ruling out impaired cell entry. Production of intracellular infectivity of the p7-QQ mutant was also impaired, although not quite to the same extent as for the generation of extracellular infectious particles which may point to a potential role of the ion channel function during virus secretion [4]. Importantly, Wozniak *et al.* reported that p7 could prevent the acidification of intracellular vesicles and, thus, might protect pH-sensitive immature intracellular virions from premature fusion during release [54]. While in this study an ion channel-defective mutant could be rescued by treatment with pharmacological inhibitors of intravesicular acidification, this was not the case for p7 deletion constructs [54] highlighting additional functions of p7 independent of its ion channel activity. In accordance with this, we observed a significant reduction of protease-resistant core protein in cells transfected with p7 mutants as compared to WT-Jc1 (Figure 2), clearly showing that virus production is arrested or impaired at a step prior to envelopment and capsid budding into the ER. Rate zonal centrifugation of detergent-free cell lysates revealed an accumulation of slow sedimenting, non-infectious, non-enveloped core complexes in the case of the p7 mutants (Figures 3 and 4). Interestingly, a similar profile was also obtained in the case of WT-Jc1 but only early (12 h) after transfection (Figure 5), suggesting that these structures were naturally occurring assembly intermediates that, in case of a non-functional p7, could not complete their maturation. These species disappeared 24 h post-transfection for the WT-Jc1 in favor of faster sedimenting enveloped infectious particles, indicating that capsid envelopment might be a rate limiting step early during assembly. On the contrary, time dependent emergence of these latter, enveloped, highly infectious core structures was abrogated, or delayed and incomplete for the Δp7_half_ and p7-QQ mutants, respectively. Finally, rate zonal centrifugation of Jc1-transfected cell lysates in presence of detergent also showed slow sedimenting core species (Figure 4E, fraction 3), confirming that p7 mutants accumulate core structures resembling naked capsids. Interestingly, the production of enveloped virus could be partially rescued for the Δp7_half_ mutant when providing functional p7 in *trans* leading to the two-peak phenotype observed for the p7-QQ mutant (Figure 5B).

Secondly, the accumulation of non-enveloped core protein structures for p7 mutants correlated with an enrichment of core protein at the surface of lipid droplets as shown by immunofluorescence analysis and cellular fractionation (Figure 6). These results suggested that, in absence of functional p7, capsid assembly takes place at lipid droplets but recruitment back to the ER and subsequent budding are disturbed. These observations confirm and complement recent results obtained by Boson *et al.* who reported that p7 expression alone induces a redistribution of core from lipid droplets to the ER [61]. This was modulated by NS2 in a genotype-dependent manner and unrelated to p7 ion channel function. However, the authors only expressed individual HCV proteins and did not investigate p7 mutants in the context of virus infection where a more complex interaction pattern between viral proteins likely occurs. Notably, several independent groups have recently shown that NS2 establishes a number of protein-protein interactions required for HCV assembly [23], [43], [44], [45], [46]. A complex consisting of NS2, NS3, NS5A and the E1–E2 envelope glycoproteins was shown to form at the ER in proximity to the LDs [45]. Although data for the localization of p7 in infected cells are missing, the polypeptide is able to interact with NS2 [43], [45] and could therefore belong to the same complex. Since NS5A interacts with core at the surface of lipid droplets [10], [25], [41], it might recruit the whole complex to assembling capsids, thereby facilitating envelopment. While p7 was only assigned a subsidiary role of regulating NS2 protein interactions [45], [46] possibly by modulating its topology [44] or stability [46], the results of Boson *et al.* clearly show that it affects core localization independently of NS2 indicating a putative direct or indirect core-p7 interaction between the two proteins and additional functions of p7 during assembly.

Strikingly, cells transfected with the p7-QQ mutant showed an enrichment of high-order core complexes mostly ranging in size from 480 kDa to over 1,000 kDa (Figure 7C and 7D) compared to WT-Jc1. Moreover, particularly in the separation of core structures by native, gradient electrophoresis, we observed clearly more core protein signal for p7-QQ (Figure 7C). It is possible that the dominant core species present in cells transfected with WT-Jc1 was too large to enter the polyacrylamide gel as would be expected for fully-assembled capsids. Alternatively, antibody recognition of core present in native protein complexes may be impaired which could result in the observation of less core protein signal for WT-Jc1 compared to the p7-QQ mutant (Figure 7C). Since we normalized the samples prior to loading for equal protein content by a core-specific ELISA, we can exclude that differential abundance of core protein is responsible for this observation. In contrast, these findings suggest that in the mutant either more core protein species were able to enter the gel or they were better detectable by the antibody, thus arguing that the mutant p7 protein altered capsid assembly. This conclusion is also corroborated by our rate zonal centrifugation assay performed in presence of detergent. Indeed, compared with WT-Jc1 and core-C69-72A, the p7 mutants showed an enrichment in fast sedimenting core complexes (Figure 7A, fraction 5) which poorly protected RNA from enzymatic digestion (Figure 7B). In contrast, the dominant core species of WT-Jc1 present in fraction 3 efficiently protected RNA from RNase digestion unless the shielding capsid was previously removed by protease treatment. Hence, mutation in p7 caused an accumulation of high-order core complexes which might be loosely associated with but have not yet completely incorporated the viral RNA. Whether those core complexes represent capsid assembly intermediates or aberrant core aggregates remains to be investigated but the results clearly point to a role of p7 during RNA encapsidation.

To get a better understanding of the phenotypes observed for the p7 mutants, we compared them to structural protein mutants, namely the core-C69-72A, E1-K179Q and ΔE1E2 mutants. These point and deletion mutations reduced virus titers to levels comparable to the p7-QQ and Δp7_half_ mutations, respectively, indicating that differences in phenotypes were not due to varying potencies of the mutations but rather pointed to distinct mechanisms of action. The core mutant core-C69-72A was first described by Murray *et al.* who used an alanine scanning approach to identify residues important for virus production [56]. This mutation inhibited assembly of infectious virus particles without altering the protein stability or its association with LDs [56]. We confirmed those observations in the Jc1 context and further characterized the mutant assembly defect. Similarly to p7-QQ mutant, this core mutant showed an accumulation of naked capsid-like core complexes at the surface of LDs. However, no impairment of capsid assembly, as far as it could be resolved in our assays, was observed. Thus, this mutation seemed to interfere with budding via a mechanism of action different from the p7 mutations. Interestingly, cells transfected with the core mutant often exhibited large lipid droplet aggregations at the cell periphery (data not shown) contrasting with the perinuclear concentration typically seen for the p7 mutants and for most HCV genotypes, or with the diffuse localization pattern observed in Jc1-transfected cells. A correlation between inefficient virus assembly and core accumulation at LDs has been previously reported [60]. However, core accumulation at the LD surface typically displaces ADRP leading to a redistribution of LDs towards the MTOC (microtubule organization center) in the perinuclear region [64]. In case of the C69-72A mutant, LDs seemed to be relocalized in the opposite direction, thereby increasing the distance between sites of capsid assembly and the ER budding site, which could explain the impairment in capsid envelopment. Interestingly, Murray *et al.* reported a rescue of infectious virus production by two independent second site mutations, one in the first TM segment of p7, the other one in the third TM segment of NS2, supporting the notion that p7 and NS2 are important for budding of the capsid into the ER [56].

Residue K179 in E1 has been shown to be important for heterodimerization of the viral glycoproteins [57] and its substitution with a glutamine residue leads to a reduced E1/E2 interaction [46] and a strong decrease in infectious virus production [4]. Surprisingly, we found that extracellular core levels remained nearly unchanged (Figure 1C) suggesting that only the specific infectivity of virus particles was affected and not particle assembly and release *per se*. Accordingly, E1-K179Q behaved as WT-Jc1 in all subsequent characterization assays. Hence, efficient heterodimerization of the viral glycoproteins is no prerequisite for particle assembly and envelopment but essential for subsequent entry steps into new cells [65], [66].

**Figure 8:**
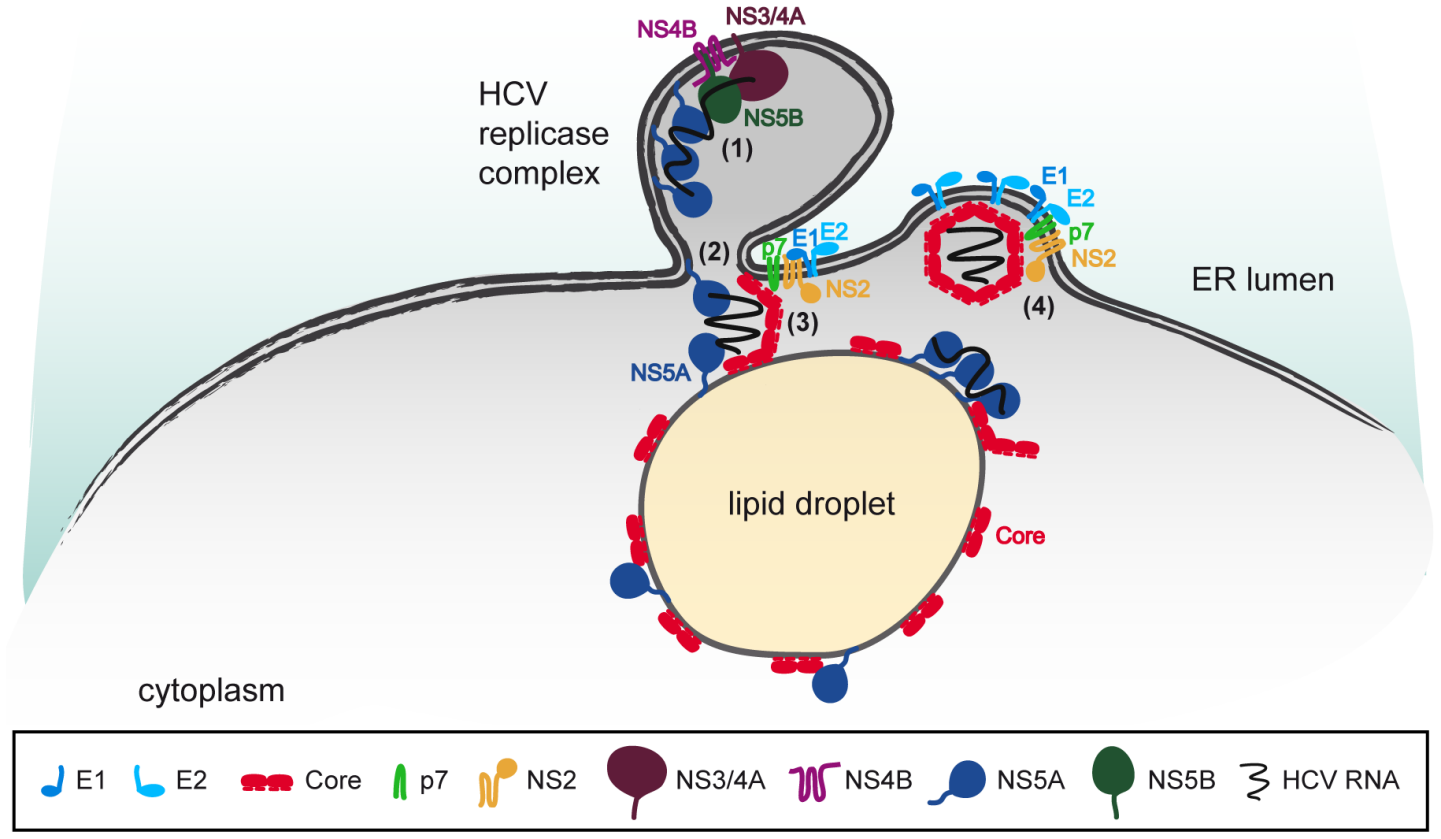
Proposed HCV assembly model. Upon replication of the viral RNA (1), the newly synthesized genome is delivered by NS5A to the core protein residing at the surface of lipid droplets (2). The presence of RNA most likely triggers initial assembly of high-order capsid-like core complexes (3), however, only the membrane scaffold provided during budding, which is initiated by p7 and the glycoproteins, allows completion of capsid assembly (4).

To further analyze possible contributions of the viral glycoproteins in HCV assembly, we created a deletion mutant lacking both E1 and E2. Not surprisingly, envelopment of capsids was completely abrogated as seen by rate zonal centrifugation (Figure 3) pointing towards a role of the glycoproteins during the budding process. This supports the model of Popescu *et al.* where E2 is part of the protein interaction complex ultimately leading to virion assembly [45]. This argues that the glycoproteins, just like p7, are necessary not only for envelopment but also for the final stages of capsid assembly. One possible explanation for this link between envelope glycoproteins and capsid assembly could be that, due to the high hydrophobicity of core domain 2, the membrane scaffold provided by the ER during the budding process is needed for the formation of fully-closed capsids. Thus, we propose the following model of HCV assembly (Figure 8): HCV RNA is translocated from the replicase complex to lipid droplets by NS5A through interaction with core. In the presence of viral RNA, assembly of high-order core complexes is initiated. The bridging function of NS2 between the glycoproteins and replicase components provides a close proximity of replication and assembly sites while p7 might directly or indirectly recruit the core assembly intermediate to the ER membrane, thereby providing the scaffold necessary for completion of capsid assembly and initiating the budding process. However, several open questions remain. Is there a direct interaction between core and p7? Which other proteins does p7 interact with? Is the ion channel activity involved? Is p7 part of the virus particle and does it have a role during cell entry? These are interesting questions to be resolved in the future.

## Materials and Methods

### Cell culture

Huh-7.5 cells [67], the Huh-7.5-derived stable cell line expressing core, E1, E2, p7 and NS2 of the J6CF isolate [59], and Huh7-Lunet-hCD81-Gluc cells [68] were grown at 37°C and with 5% CO_2_ in Dulbecco's modified Eagle's medium (DMEM; Invitrogen, Karlsruhe, Germany) supplemented with 2 mM L-glutamine, non-essential amino acids, 100 U/ml of penicillin, 100 µg/ml of streptomycin, 10% fetal calf serum, and for the latter two 5 µg/ml of blasticidin. Note that Huh7-Lunet-hCD81-Gluc cells are derived from the highly permissive Huh7-Lunet-hCD81 cells [69] via lentiviral gene transfer to express the secreted gaussia luciferase which is a simple biomarker to assess cell density and viability.

### Plasmids

The plasmids pFK-Jc1, pFK-Jc1-Δp7_half_, pFK-Jc1-p7-KR33,35QQ (p7-QQ mutant) and pFK-Jc1-E1-K179Q have been previously described [4], [58]. The pFK-Jc1-core-C69-72A plasmid which carries 4 consecutive alanine substitutions of core residues 69-72 [56] was created via PCR mutagenesis. Plasmid pFK-Jc1-ΔE1E2 which carries a complete deletion of the E1–E2 coding region encompassing residues 192 to 750 of the J6CF [70] isolate was created by a PCR-based strategy. Likewise, construct pFK-Jc1Δcore was created by PCR-based mutagenesis. It encodes a Jc1 derivative that has an in frame deletion encompassing residues 17 through 131 of the core coding region. Finally, to express core protein in the absence of E1, E2, p7 and NS2, we created a derivative of the bicistronic JFH1 replicon pFK PI-EI-NS3-5B/JFH1 [71] expressing J6CF-derived core from the first cistron and designated pFK PI-Core/J6-EI-NS3-5B/JFH1. Each construct was verified by sequencing. Additional information including the sequences of these constructs is available upon request.

### In vitro transcription, electroporation and quantification of viral core, RNA and infectious titers

Methods for in vitro transcription of HCV RNA and its electroporation into Huh-7.5 cells have been previously reported [72]. HCV core protein and RNA were quantified respectively by ELISA [68] and qRT-PCR [72]. Intra- and extracellular infectious titers were determined as previously described [68].

### Protein electrophoresis and immunoblotting

For experiments under denaturating conditions, protein samples were obtained from scraped cells lysed directly in SDS sample buffer (final concentrations (1×): 75 mM Tris-HCl pH 6.8; 0.6% (v/v) SDS, 15% (v/v) glycerol, 0.001% (w/v) bromophenol blue; 7.5% (v/v) ß-mercaptoethanol) for 10 min at 95°C and separated by SDS-PAGE. Immunoblotting was performed essentially as described before [72].

For blue-native PAGE, cells were harvested and lysed using the NativePAGE Sample Prep kit (Invitrogen, Karlsruhe, Germany) with 1% n-dodecyl beta-D-maltoside (DDM, AppliChem, Darmstadt, Germany) under non-reducing conditions without heating according to the manufacturer's recommendations. Typically, 1×10^6^ HCV-transfected Huh7-Lunet-hCD81-Gluc cells were lysed in 100 µl volume, of which 25 µl were loaded onto 4–16% or 3–12% polyacrylamide gradient gels (Invitrogen) and electrophoresis was performed at a constant voltage of 150 V. Proteins were transferred on a PVDF membrane using a dry blotting system (IBlot, Invitrogen).

For two-dimensional resolution, individual lanes from blue-native PAGE were cut out of the gel, equilibrated in 2× SDS sample buffer for 30 min, stacked peripendicular above a 11% polyacrylamide SDS gel in one large stacking gel pocket and overlayed with 1× SDS sample buffer. Gel electrophoresis and immunoblotting were performed as above [72].

For western blotting, the following antibodies were used: anti-core (C7-50; kindly provided by D. Moradpour; [73]; diluted 1∶1,000), anti-ADRP (adipose-differentiation related protein, AP125 antibody; Progen, Heidelberg, Germany; diluted 1∶200), anti-Calreticulin (Stressgen, Victoria, BC, Canada; diluted 1∶2,000) and anti-mouse-HRP (Sigma, Steinheim, Germany; diluted 1∶20,000).

### Proteolytic digestion protection assay

HCV-transfected cells seeded in 6-well dishes were scraped in 170 µl proteinase K buffer (50 mM Tris-HCl pH 8.0; 10 mM CaCl_2_; 1 mM DTT) 48 h post-transfection and subjected to five cycles of freeze and thaw. Subsequently, 50 µl of the crude lysate was left untreated, while 50 µl was treated with 50 µg/ml proteinase K (Roche, Mannheim, Germany) for 1 h on ice and another 50 µl was lysed with 5% (v/v) Triton X-100 prior to proteinase K treatment. Proteinase K digestion was terminated by addition of PMSF (phenylmethylsulfonyl fluoride; AppliChem) at a final concentration of 5 mM and incubation of the sample on ice for 10 min. Subsequently, 13 µl of 5× SDS sample buffer were added and the sample was heated to 95°C for 10 min. The amount of residual core protein was determined by SDS-PAGE and immunoblotting. Alternatively, to quantify core amounts by ELISA, proteinase K was inactivated with 10 mM PMSF, heating to 95°C and addition of 50 µl 2× protease inhibitor cocktail (Roche, Mannheim, Germany).

### RNase digestion protection assay

Three 50-µl samples from individual 1-ml gradient fractions were either (i) left untreated, (ii) treated with RNase A (Sigma) (50 µg/ml for 4 h at RT) or (iii) pretreated with proteinase K (5 µg/ml, for 1 h on ice; reaction was stopped by the addition of 10 mM PMSF and 5 µl of 10× protease inhibitor cocktail) prior to RNase A digestion (as above). Subsequently, total RNA was extracted using the NucleoSpin RNAII kit (Macherey-Nagel, Düren, Germany) according to the manufacturer's instructions and HCV RNA quantified by qRT-PCR as previously described [72].

### Rate zonal centrifugation

HCV-transfected cells seeded in 10-cm dishes were scraped in 250 µl TNE buffer (10 mM Tris-HCl pH 8.0; 150 mM NaCl; 2 mM EDTA) 48 h post-transfection and subjected to five cycles of freeze and thaw. Postnuclear supernatants collected after centrifugation of the crude lysate for 5 min at 4°C and 1,700× g were layered on top of preformed continuous 0–30% sucrose/TNE gradients and spun at 270,000× g for 1 h at 4°C in a Sorvall TH-641 rotor. Subsequently, 10 fractions (1 mL each) were harvested from the top and the respective refraction index was measured by refractometry. For rate zonal centrifugation in the presence of detergent, 1% DDM was added to the sucrose solutions and to the postnuclear supernatant.

### Lipid droplet fractionation

Lipid droplets were isolated from HCV-transfected cells following a previously published protocol [74]. Briefly, 48 hours post-transfection, pellets of HCV-transfected cells were harvested from 15-cm dishes by scraping and resuspended in 1 ml ice-cold HLM (hypotonic lysis medium; 20 mM Tris-HCl pH 7.4; 1 mM EDTA) followed by incubation on ice for 10 min and lysed by 6–8 strokes in a Potter-Elvehjem tissue homogenizer. The postnuclear supernatant was mixed with one third volume of HLM containing 60% sucrose and layered under 5 ml HLM containing 5% sucrose and 5 ml sucrose-free HLM. Rate zonal centrifugation was performed at 28,000× g for 30 min at 4°C in a Sorvall TH-641 rotor. The uppermost fractions enriched in lipid droplets were collected and the purification process was controlled by SDS-PAGE and immunoblotting of ADRP and calreticulin in the isolated fractions.

### Indirect immunofluorescence analysis

Indirect immunofluorescence staining was performed as described before [72]. HCV core protein was detected using the mouse monoclonal C7-50 antibody diluted 1∶1,000 in PBS supplemented with 5% goat serum (Sigma) followed by a secondary antibody specific for murine IgG conjugated with Alexa-Fluor 568 (Invitrogen) at a dilution of 1∶1,000. For LD detection, BODIPY (493/503) (Invitrogen) was added to the secondary antibody at a dilution of 1∶1,000. Cell nuclei were counter-stained for 1 min at RT with DAPI (Invitrogen) diluted 1∶3,000.

### Statistical analysis

Data were analyzed statistically in R (http://www.r-project.org). Normality of data was assessed using histograms and qq-plots. Differences between sample populations were assessed using a two-sample Welch's t-test. P-values were calculated and statistical significance reported as highly significant with (***) if p≤0.001, (**) if p≤0.01, and significant (*) if p≤0.05. Differences were considered not significant (n.s.) for p>0.05.

## Supporting Information

Figure S1 **Characterization of differentially-sedimenting core complexes in cells transfected with a replicon expressing core.** Lysates of cells transfected with Jc1-WT or a subgenomic replicon expressing J6CF core protein in the first cistron (in the absence of E1, E2, p7 and NS2) were separated by rate zonal density gradient centrifugation. Each fraction was analyzed core protein content by ELISA.
(TIF)

Figure S2 **Characterization of differentially-sedimenting RNA complexes.** Lysates of cells transfected with Jc1-WT, Jc1-Δcore or a subgenomic replicon expressing core protein (core replicon) were separated by rate zonal density gradient centrifugation. Each fraction was analyzed for HCV RNA copy numbers by qRT-PCR. Note that deletion of the core coding region in Jc1-Δcore compromises replication fitness (data not shown). This likely explains consistently lower levels of viral RNA observed in cells transfected with this mutant.
(TIF)

Figure S3 **HCV RNase digestion protection assay of cell lysates from wild type and mutant HCV genome transfected cells.** Cells were transfected with given viral constructs and harvested 48 h post-transfection. Cell lysates were subjected to rate zonal density gradient centrifugation in the presence of non-ionic detergent (1% DDM). Fractions 3 to 5 of the gradients were collected and subjected to RNase digestion or left untreated. As a background control, samples were treated with proteinase K to remove protecting capsids, prior to RNase treatment. The amount of residual HCV RNA was quantified by qRT-PCR. Mean values two independent gradients including the standard deviations are given.
(TIF)
