# Supplementary figures and images for: Hepatitis C Virus p7 is Critical for Capsid Assembly and Envelopment
Source: PLoS Pathog. 2013 May 2;9(5):e1003355. doi: 10.1371/journal.ppat.1003355 (PMC3642076; doi:10.1371/journal.ppat.1003355)

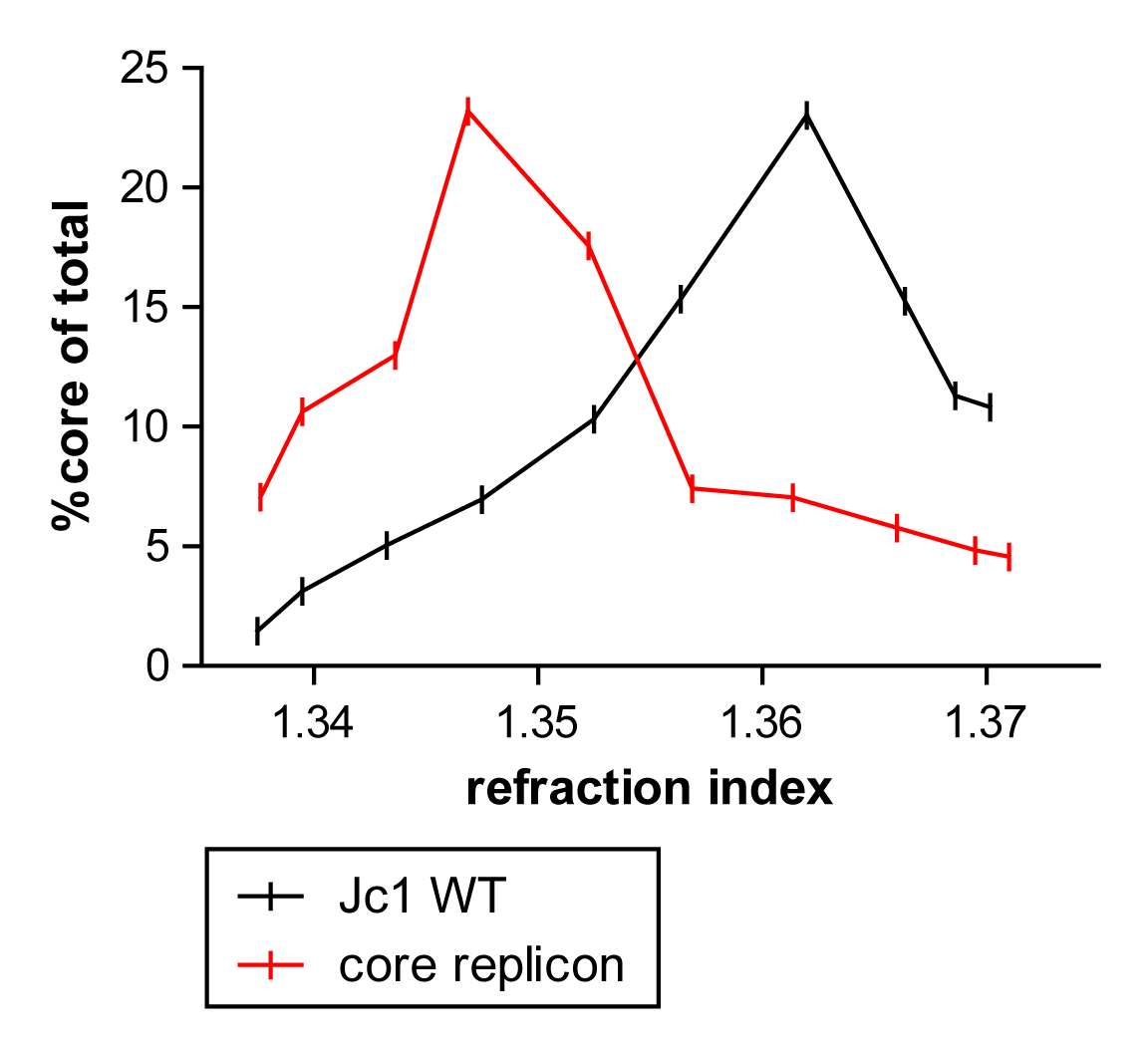

Supplement: Figure S1 — Characterization of differentially-sedimenting core complexes in cells transfected with a replicon expressing core. Lysates of cells transfected with Jc1-WT or a subgenomic replicon expressing J6CF core protein in the first cistron (in the absence of E1, E2, p7 and NS2) were separated by rate zonal density gradient centrifugation. Each fraction was analyzed core protein content by ELISA. (TIF) [file ppat.1003355.s001.tif]

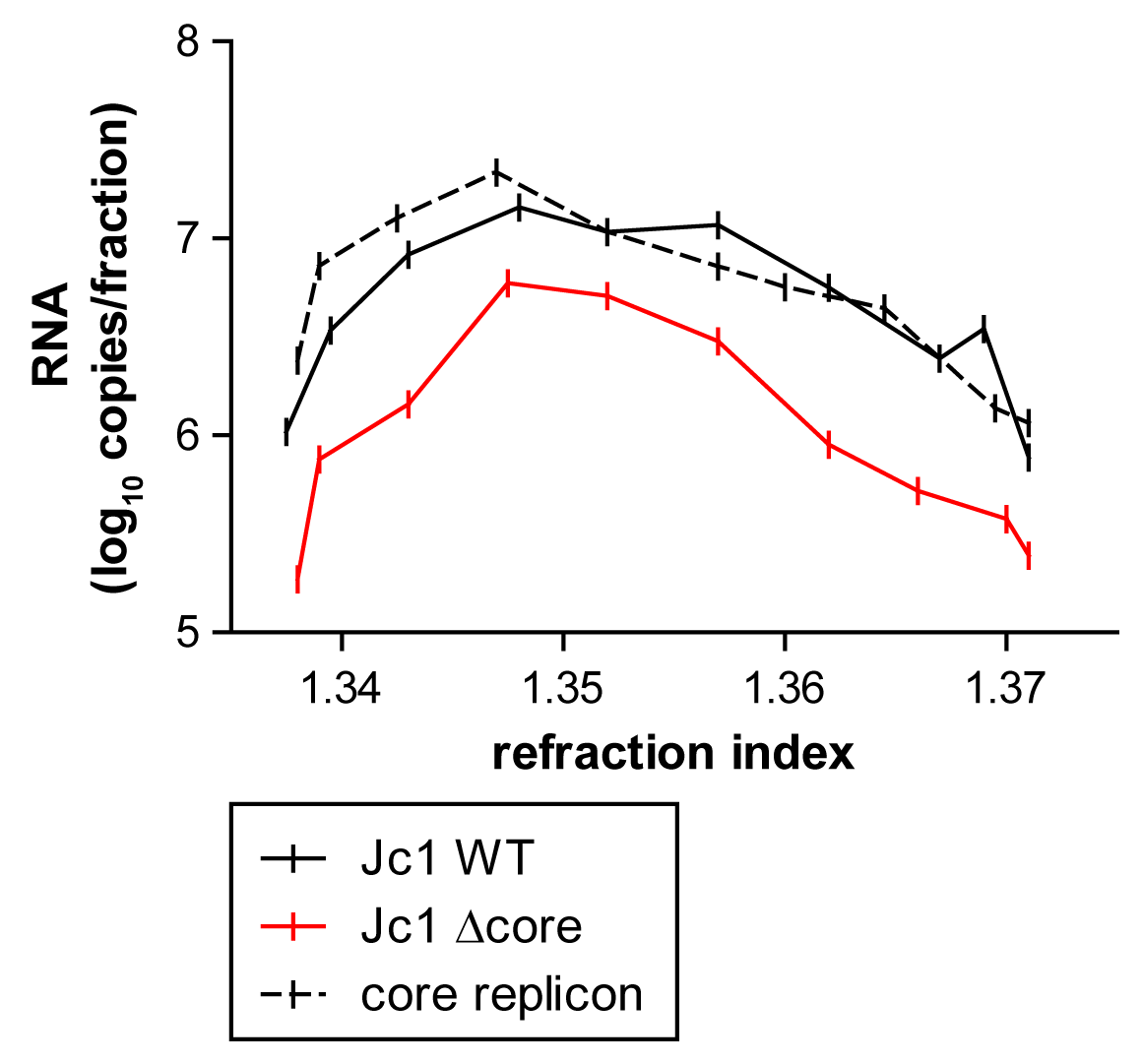

Supplement: Figure S2 — Characterization of differentially-sedimenting RNA complexes. Lysates of cells transfected with Jc1-WT, Jc1-Δcore or a subgenomic replicon expressing core protein (core replicon) were separated by rate zonal density gradient centrifugation. Each fraction was analyzed for HCV RNA copy numbers by qRT-PCR. Note that deletion of the core coding region in Jc1-Δcore compromises replication fitness (data not shown). This likely explains consistently lower levels of viral RNA observed in cells transfected with this mutant. (TIF) [file ppat.1003355.s002.tif]

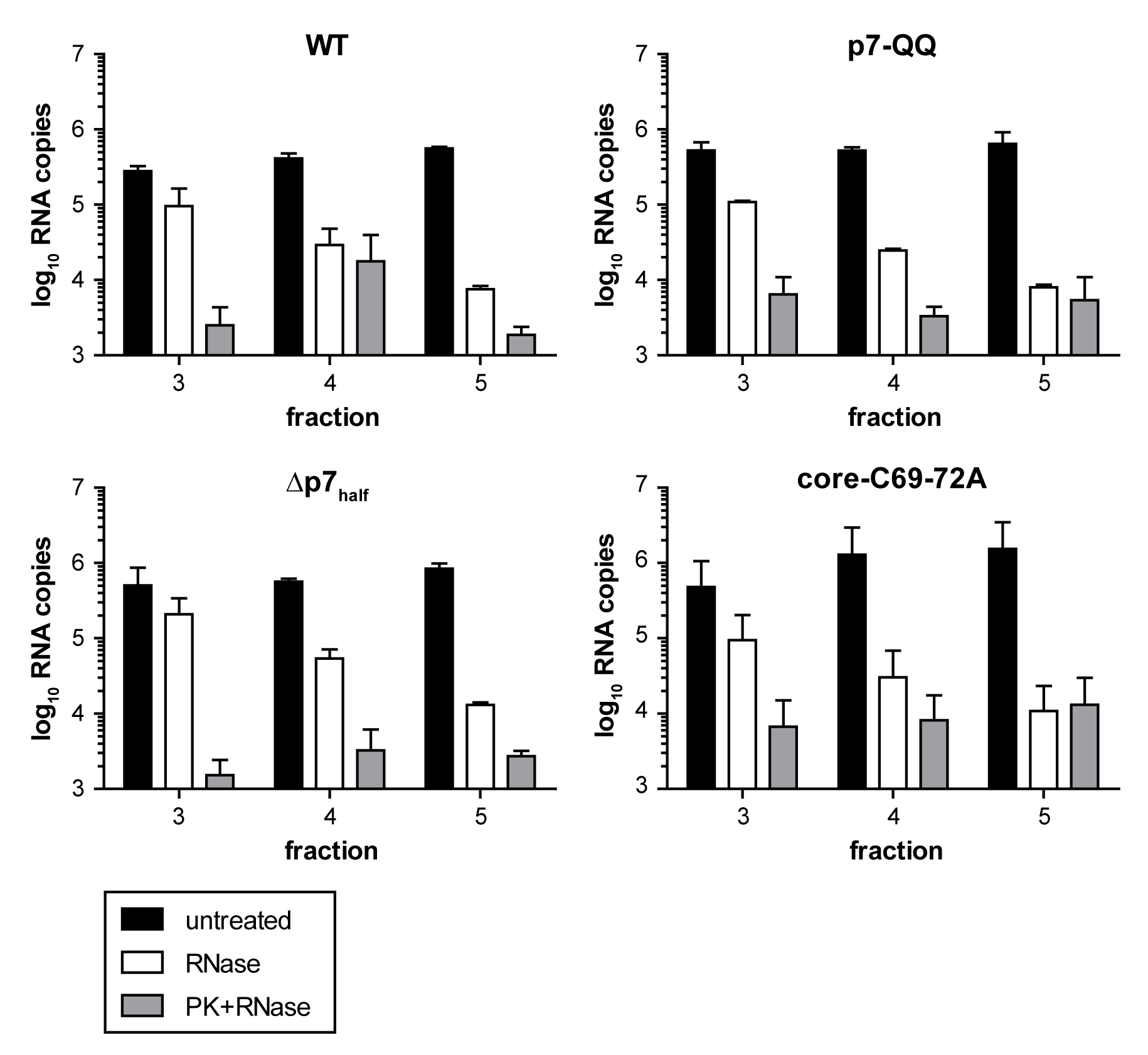

Supplement: Figure S3 — HCV RNase digestion protection assay of cell lysates from wild type and mutant HCV genome transfected cells. Cells were transfected with given viral constructs and harvested 48 h post-transfection. Cell lysates were subjected to rate zonal density gradient centrifugation in the presence of non-ionic detergent (1% DDM). Fractions 3 to 5 of the gradients were collected and subjected to RNase digestion or left untreated. As a background control, samples were treated with proteinase K to remove protecting capsids, prior to RNase treatment. The amount of residual HCV RNA was quantified by qRT-PCR. Mean values two independent gradients including the standard deviations are given. (TIF) [file ppat.1003355.s003.tif]
